# India’s rainfed sorghum improvement: Three decades of genetic gain assessment for yield, grain quality, grain mold and shoot fly resistance

**DOI:** 10.3389/fpls.2022.1056040

**Published:** 2022-12-19

**Authors:** Mallela Venkata Nagesh Kumar, Vittal Ramya, Mahalingam Govindaraj, Appavoo Dandapani, Setaboyine Maheshwaramma, Kuyyamudi Nanaiah Ganapathy, Kosnam Kavitha, Manthati Goverdhan, Rumandla Jagadeeshwar

**Affiliations:** ^1^ Professor Jayashankar Telangana State Agricultural University, Rajendranagar, Hyderabad, Telangana, India; ^2^ HarvestPlus program, The Alliance of Bioversity International and the International Center for Tropical Agriculture (CIAT), Cali, Colombia; ^3^ Indian Council of Agricultural Research-National Academy of Agricultural Research Management, Rajendranagar, Hyderabad, Telangana, India; ^4^ Indian Council of Agricultural Research -Indian Institute of Millets Research Rajendranagar, Hyderabad, Telangana, India

**Keywords:** sorghum, landraces, dual-purpose, grain yield, fodder yield, grain mold, shoot fly, genetic gain

## Abstract

Sorghum is a climate-resilient cereal and staple food crop for more than 200 million people in arid and semi-arid countries of Asia and Africa. Despite the economic importance, the productivity of sorghum in India is constrained by biotic and abiotic stresses such as incidences of shoot fly, grain mold and drought. Indian sorghum breeding focused on dual-purpose (grain and fodder), short-duration varieties with multiple resistance/tolerance to pests and diseases and improved nutritional quality (high protein, iron and zinc and low fat). In this context, it is important to ascertain the genetic progress made over 30 years by assessing the efficiency of past achievements in genetic yield potential and to facilitate future genetic improvement. The current study determined the genetic gain in 24 sorghum varieties developed by the national and state level research systems during 1990-2020. The 24 varieties were evaluated for three years (2018-2020) at six locations in Telangana state for yield, nutritional characteristics and tolerance to shoot fly and grain mold. The absolute grain yield genetic gain from the base year 1990 is 44.93 kg/ha/yr over the first released variety CSV 15. The realized mean yield increased from 2658 kg/ha of the variety CSV 15 in 1990s to 4069 kg/ha of SPV 2579 developed in 2020s. The absolute genetic gain for grain mold resistance is -0.11 per year with an overall relative gain of 1.46% over CSV 15. The top varieties for grain yield (SPV 2579, SPV 2678 and SPV 2578), fodder yield (PYPS 2, SPV 2769 and SPV 2679), shoot fly tolerance (PYPS 8, PYPS 2 and SPV 2179), mold tolerance (PYPS 8, PYPS 2 and SPV 2579) and high protein (PYPS 8, PYPS 2 and SPV 2769) were identified for possible scale up and further use in breeding program diversification. The study revealed that sorghum varieties bred with diverse genetic backgrounds such as landraces and with tolerance to pests and diseases had stable yield performance. Application of genomics and other precision tools can double genetic gains for these traits to strengthen sorghum cultivation in rainfed areas serving food and nutrition security.

## Introduction

Sorghum (*Sorghum bicolour* L. Moench) is an important crop in the semi-arid and arid regions of South Asia and sub-Saharan Africa that are subjected to frequent droughts, low and erratic rainfall and high mean temperature (Reddy and Reddy, 2019). Sorghum is cultivated in 40.25 million ha in the world with a production of 58.70 million tonnes and productivity of 1458 kg/ha (FAO, 2021). The top 10 sorghum producers, the USA, Sudan, Mexico, Nigeria, India, Niger, Ethiopia, Australia, Brazil and China, contribute about 77% of world sorghum production (Aruna and Cheruku, 2019). It is the dietary staple for more than 200 million people in these regions and is a source of food and fodder, especially in the traditional, small-holder farming sector (Visarada and Aruna, 2019). Sorghum is considered as “healthy cereal” and is a good source of carbohydrates (68%), proteins (10%), micronutrients and phytochemicals with nutraceutical properties (Visarada and Aruna, 2019). The per capita consumption of sorghum is high at 75 kg/year in major sorghum growing regions in India and it contributes to more than 50% of the iron and zinc requirement in low-income group populations in India (Rao et al., 2006; Ashok kumar et al., 2011; Ashok kumar et al., 2013).

Sorghum is a climate-smart C_4_ crop with the ability to produce grain and fodder in harsh environments under low input conditions with high net returns (Hao et al., 2021). It is resilient to diverse environmental conditions with a forte to perform well in marginal conditions under water and temperature constraints without competing with other food crops (Griebel et al., 2019). Keeping in view the growing demand for limited freshwater source, growing usage of marginal farmland and changing climatic patterns, sorghum has a vital role to play in ensuring nutritional security of the world and more so in the context of ever-decreasing arable land and water (Deshpande et al., 2016) and frequent occurrences of environmental excesses like floods, water inadequacy and temperature extremes (Mickelbart et al., 2015).

India is the second largest country occupying 13.6% of the sorghum cultivated area after Nigeria (14.16%). However, the productivity is very low at 1250 kg/ha compared to that of countries like China and USA at 4854 and 4594.6 kg/ha, respectively (FAO, 2021). Furthermore, the yield is also far less than the attainable yield of 3500 kgha^-1^under good management practices. The low yield of sorghum is attributed to a wide array of reasons *viz.*, lack of compatible improved varieties suitable for different agro-ecological zones, the incidence of abiotic and biotic stresses and use of traditional varieties and conventional production practices. Drought (rainfed sorghum), cold (post-rainy season sorghum) and problematic soils (soil salinity and Al toxicity) are important abiotic constraints. It is estimated that about 32% of the sorghum crop in India is lost due to the incidence of insect pests during the rainy season (Borad and Mittal, 1983) and 26% during the post-rainy season (Daware et al., 2012). Sorghum shoot fly [*Atherigona soccata* (Rond.)] is among the most destructive pests in sorghum causing most damage during the seedling stage. Grain mold is the most important disease and causes yield losses ranging from 30 to 100% depending on the cultivars and weather conditions (Singh and Bandyopadhyay, 2000).

Systematic research for sorghum improvement in India started with the establishment of the Accelerated Sorghum and Millet Improvement Project (ASMIP) in 1962 with the objective to initiate hybrid breeding (Tonapi et al., 2011). Since the release of the first sorghum hybrid, Coordinated Sorghum Hybrid 1 (CSH 1) in 1964 (Rao, 1982), remarkable progress has been made in sorghum improvement by diversifying the parental lines for yield, maturity, pest tolerance and quality by utilizing indigenous and exotic germplasm (House et al., 1996) resulting in the development and release of several hybrids (3.0-4.2 t/ha) and varieties (2.8-3.8 t/ha) in the next five decades. Since the 1990s, with increasing incidence of shoot fly and grain mold, sorghum breeding was re-oriented towards the development of dual-purpose varieties with high yield and tolerance to these two stresses. In Advanced Evaluation Trials (AET), it became mandatory that the entries are screened at the regional centres for tolerance to shoot fly and grain mold in addition to high yields before release at the national level. At present, sorghum research is being carried out at the Indian Council of Agricultural Research-Indian Institute of Millets Research (ICAR-IIMR) in collaboration with the All India Co-ordinated Research Program on sorghum (AICRP on Sorghum) with 21 centres distributed across 10 states of India, and the International Crop Research Institute for Semi-Arid Tropics (ICRISAT). To date, the coordinated efforts have led to the release of 35 hybrids and 30 varieties in grain, forage and sweet sorghum types (Aruna and Cheruku, 2019).

Sorghum yield in India has increased considerably from 969 kg/ha in 1990-91 to 1210 kg/ha in 2020-21 (FAO, 2021). Though these on-farm annual yield estimates provide useful information on trends in sorghum performance, it should be noted that they are a result of both plant breeding and agronomic practices (Pfeiffer et al., 2018) and do not necessarily reflect the improvement due to the efficiency of past breeding efforts alone in sorghum and hence may not provide precise information with the genetic progress made in improving the yield. The demand for sorghum grain has been on a consistent rise (Boyles et al., 2016) creating importance for grain yield but the progress has been slower in comparison to other cereals including maize and rice (Mason et al., 2008). Sorghum needs 15% gain in yield to be more competitive (Aruna and Cheruku, 2019) for which it is important to review the past yield gains to predict future gains from selection and assess the progress of past and present selection strategies. In this context, it is important to evaluate sorghum genotypes developed in different eras under equal environment and management conditions to establish the relative value of plant breeding efforts in yield gains (Smith et al., 2004; Duvick, 2005).

There are extensive studies estimating the genetic gains achieved in major crops with the gains varying with crop, country and period. Genetic yield gain computed in rice ranged from non-significant (Muralidharan et al., 2019) to significant positive gain (0.68% under irrigated control; 0.87% under moderate reproductive stage drought stress; 19% severe reproductive stage drought stress) (Oluwaseun et al., 2022) for grain yield. In maize, positive genetic gains for grain yield were estimated to have increased by 109.4 kg/ha/yr (optimal conditions), 32.5 kg/ha/yr (managed drought), 22.7 kg/ha/yr (random drought), 20.9 kg/ha/yr (low nitrogen) and 141.3 kg/ha/yr (maize streak virus) (Masuka et al., 2017); In potato, the annual genetic gains for tuber yield were small at Nordic region of Europe (0.3% per year) and Sweden (0.7% per year) (Ortiz et al., 2022). In soybean, positive genetic gains for seed yield were reported in Brazil in the South Region (0.33 to 0.42% per year) and Midwest region (0.47 to 0.77% per year) (Milioli et al., 2022).

Several studies on the genetic gain in sorghum have been reported across the world in the regions of United States (Smith and Frederiksen, 2000; Pfeiffer et al., 2018), Australia (Stephens et al., 2012), Argentina (Gizzi and Gambin, 2016), Mali (Rattunde et al., 2016), Ethiopia (Chala et al., 2019), Haiti (Muleta et al., 2019). However, compared to other crops, there are far fewer studies examining the changes occurring due to long-term selection within sorghum breeding programs in India. To our knowledge, there is only one study conducted by Rakshit et al. (2014) estimating the genetic gain for grain yield over years in the Indian sorghum improvement program. The study used a historical set of data on the performance of genotypes from 1970 to 2009 and found that the genetic gain was prominent in rainy-season hybrid trials (18.5 kg/ha/yr) whereas it was insignificant in post-rainy season hybrid and varietal trials.

The sorghum breeding programme in India which was initiated to develop hybrids was re-oriented during the 1990s to develop dual-purpose varieties with tolerance to shoot fly and grain mold to enhance the yield gains (Das et al., 2020). While the increase in sorghum yield from 1991 to 2020 was estimated at 25%, there have been no reports on the accelerated genetic gain in breeding progress of grain mold and shoot fly tolerant sorghum varieties generated during the last three decades, thus making it difficult to completely ascertain the genetic gain that has been made in grain yield in relationship to grain mold and shootfly tolerance in sorghum varieties that have been developed and released in India. Therefore, the current study was conducted with the following objectives (i) to determine the genetic gain for grain yield and fodder yield of sorghum with tolerance to shoot fly and grain mold, (ii) to measure changes in stress-tolerant traits that have accompanied changes in grain yield, (iii) to identify high yielding, protein-rich varieties with yield stability for commercialization in India.

## Materials and methods

### Plant material and environments

A total of 24 sorghum varieties were evaluated in this study (**Table 1**). Majority of the varieties were developed by using the pedigree method of selection where one variety is usually selected based on its proven performance and the other to complement the first variety. Selection of individual plants was continued from F_2_ to F_4_/F_5_ until the population reached near homozygosity, after which selection was practiced among the families. At F_6_ stage, the varieties were screened separately for insect pest or disease tolerance in addition to grain and fodder yields.

**Table 1:** Description of sorghum varieties included in the study.

S. No.	Sorghum Variety	Pedigree	Stage of release/evaluation	Year of release/evaluation	Distinguishing features
1	PSV-1	MS8271 x IS3691	Released (State)	1990	Best suitable under rainfed situations; Drought tolerant, pearly white grains with good roti (flat bread) making quality
2	Palem-2	SPV86 x GD57904	Released (State level)	1998	High dry fodder yield
3	CSV-15	SPV 475 x SPV 462	Released (National)	2005	High yielding dual purpose
4	PSV-56	CSV15 x PVK801	Released (State)	2010	Dual purpose with grain mold tolerance
5	SPV 2110	Palem 2 x IS 48592	AET-II	2011	Dual purpose with grain mold tolerance
6	SPV 2122	SPV462 x SPV1329	Released (National)	2011	High yielding dual purpose with grain mold tolerance; good roti making quality
7	SPV 2178	CSV15 x SF94006	AET-I	2012	Dual purpose with shoot fly tolerance
8	SPV 2179	SPV462 x ICSV18551	AET-I	2012	High grain and fodder yield, with drought and shoot fly tolerance
9	SPV 2242	SPV 504 x ICSR103	AET-I	2013	Dual purpose with grain mold tolerance
10	SPV 2243	ICSR 90003 x IS 2394	AET-I	2013	Dual purpose with grain mold tolerance
11	SPV 2121	CSV15 x IS 22149	AET-II	2014	Dual purpose with grain mold tolerance
12	SPV 2293	CSV 15 x SF 94006	Released (State)	2014	High grain and fodder yield, tolerance to shoot fly and grain mold
13	SPV 2294	ICSR 90003 x IS 2394	AET-I	2014	High fodder yield and tolerance to grain mold and shoot fly with
14	SPV 2437	SPV86 x ICSR89064	Released (National)	2016	High yielding dual purpose variety with tolerance to grain mold and shoot mly
15	SPV 2438	SPV504 x ICSR103	AET-II	2016	Dual purpose with grain mold tolerance
16	SPV 2502	SPV462 x GD 8695	AET-I	2017	Dual purpose with drought and grain mold tolerance; good roti making quality
17	SPV 2578	ICSR90003 × IS 2394	AET-I	2018	Dual purpose with shoot fly tolerance
18	SPV 2579	PSV15 × SPV462	AET-I	2018	High yielding, drought tolerance and grain mold tolerance
19	SPV 2678	SPV878 × NJ2313	AET-I	2019	Suitable for late planting; drought tolerance and high fodder yield
20	SPV 2679	SPV462 × IS33751	AET-II	2019	Dual purpose with grain mold tolerance
21	SPV 2769	ICSR 90003 x IS 18369	AET-I	2020	High yielding, shoot fly and grain mold tolerance
22	SPV 2770	PSV 319 x PSV 407	AET-II	2020	Dual purpose with grain mold tolerance
23	PYPS 2	YPS19 x YPS72	Released (State)	2020	High yielding, yellow pericarp sorghum; high fodder yield, early maturing; high consumer preference
24	PYPS 8	YPS21 x YPS 24	AET-II	2020	High yielding, early maturity, tolerance to shoot fly and grain mold; Yellowish-red colored grains with good roti making quality

The plant material included eight varieties released at state/national levels between 1990 and 2020 (**Table 1**). These were selected for their importance and features responsible for wide cultivation. Sixteen varieties which did not go through the AET I and II trials due to non-significant yield improvement over standard checks were also included in the study because of their superior characters. The varieties PYPS 2 and PYPS 8 were developed utilizing landraces with early maturity, good grain and fodder yielding qualities and high-end product consumer preference. The remaining varieties were developed using high-yielding lines and tolerant germplasm (drought, grain mold and shoot fly) obtained from ICRISAT, Hyderabad, India and ICAR-IIMR, Hyderabad, India. The seeds for conducting the trials were obtained from the Regional Agricultural Research Station, Palem, India.

The 24 sorghum varieties were tested in six locations (Palem, Nizamabad, Adilabad, Tandur, Madhira and Hyderabad) for three years (2018-19, 2019-20 and 2020-21) for grain yield, fodder yield, and tolerance to shoot fly and grain mold. Two locations, grouped based on annual rainfall, sowing window, type of soils and end product consumer preference, and tested for three years were considered as one environment. The varieties were evaluated in three environments *i.e.*, AT (Adilabad and Tandur) region, PN (Palem and Nizamabad) region and MH (Madhira and Hyderabad) region. In each location, the trials were conducted in the experimental farms of Agricultural Research Stations of Professor Jayashankar Telangana State Agricultural University, Hyderabad, India. In all the six selected locations sorghum is cultivated during the rainy season under the rainfed system (**Table 2**).

**Table 2:** Details of the 3 environments tested for evaluation of sorghum varieties from 2018-19 to 2020-21 in Telangana State, India.

Environment	Name of the location	Latitude	Longitude	Annual rainfall (mm)	Type of soil	Period of sowing	Farmers’ preference
MH	Madhira	16.9182^0^N	80.3633^0^E	1042.1	Clay loam	2^nd^ fortnight of August to 1^st^ fortnight of September	High fodder yield varieties
	Hyderabad	17.3850^0^N	78.4867^0^E	830.8	Sandy loam		
PN	Palem	16.4939^0^N	78.3102^0^E	644.9	Sandy	2^nd^ fortnight of May to 1^st^ fortnight of June	Yellow or reddish yellow colored grains; High fodder yield
	Nizamabad	18.6725^0^N	78.0941^0^E	1017.1	Sandy loam		
AT	Tandur	17.2576^0^N	77.5875^0^E	806.2	Clay	1^st^ fortnight of June to 1^st^ fortnight of July	White grains with good roti (flat bread) making quality; Dual purpose varieties
	Adilabad	19.6641^0^N	78.5320^0^E	1157.8	Clay		

### Evaluation of varieties for yield performance and screening for tolerance to shoot fly and grain mold

Each sorghum variety was planted on six rows of 5 m length plot by using between- and within-row spacing of 45 and 10 cm, respectively. Nutrient management, inter-cultivation and weed management were carried out according to the technical recommendations for the sorghum crop. During harvest, the four central rows within each plot were sampled for grain yield and fodder yield. The number of plants with dead hearts was recorded at 28 days after emergence and shoot fly damage was calculated as the percentage of dead heart incidence (Sharma et al., 2003). All the 24 sorghum varieties were also evaluated in the sorghum grain mold nursery over the three rainy seasons (June–September) in 2018 to 2020 at all six locations under natural epiphytotic conditions for grain mold evaluation. Each genotype was sown in six rows of 5 m in length during the first fortnight of June so that the grain maturity stage coincided with the periods of frequent rainfall received in the ensuing August–September, thus predisposing the crop to grain mold disease. During rain-free days, high relative humidity (>90%) was maintained from the flowering to the physiological maturity stage by using sprinkler irrigation. About 10 uniformly flowering plants with the same flowering window were tagged in each row. The visual panicle grain mold rating (PGMR) was taken on each of the tagged plants at the prescribed physiological maturity by using a progressive 1–9 scale, where 1 = no mold infection, 2 = 1–5%, 3 = 6–10%, 4 = 11–20%, 5 = 21–30%, 6 = 31–40%, 7 = 41–50%, 8 = 51–75%, and 9 = 76–100% molded grains on a panicle (Singh and Bandyopadhyay, 2000; Thakur et al., 2007). All the trials at each location were conducted in a complete randomized block design with three replications.

### Estimation of protein and total fat

Whole grains of 24 sorghum varieties were collected from the field trials at Hyderabad in 2020 and analyzed for protein and fat by standard methods of AOAC (2016). The protein level was quantified by using the generic combustion method of analysis with the LECO F-528 nitrogen analyzer (LECO, St. Joseph, MI, USA) calibrated with ethylene diamine tetra acetic acid according to the association of official analytical chemists method (AOAC, 2016). The grain samples were ground to a suitable fineness to pass No. 20 sieve and dried at 102 ± 2°C for 2 h. A moisture-free sample weighing 200 mg was analyzed to estimate protein content. The total fat content was estimated by the automatic Soxtherm extraction unit (Gerhardt Analytical Systems, Königswinter, Germany) using petroleum ether (60-80°C) as the solvent. After evaporation, the sample was dried in the hot air oven at 100°C for 1 hour, cooled in a desiccator and weighed to estimate the fat content (%).

### Statistical analyses

The statistical analyses were performed using a linear model where variety, environment and repetitions within each environment and genotype × environment interaction were considered random effects, while the main effect was considered to be fixed in the model (Alvarado et al., 2015 and Alvarado et al., 2020). In this case, all random effects were assumed to be normally distributed. The best linear unbiased predictors (BLUPs) of the varieties were obtained for each trait using a linear model according to Alvarado et al. (2015). For the analysis combining data across environments and considering the RCBD, the model was:


$$Y_{ijkl}=\;µ\;+\;Env_{i}+\;Rep_{j}\left(Env_{i}\right)\;+\;Gen_{l}+\;Env_{l}x\;Gen_{i}+\epsilon_{ijkl}$$

where *Y_ijkl_* is the evaluated trait, *μ* is the mean effect;

Env*_i_* is the effect of the *i*th environment,

Rep*_j_* (Env*_i_*) is the effect of the *_j_* th replicate within the *_i_* th environment;

Gen*_l_* is the effect of the *_l_* th genotype;

Env*_i_* × Gen*_l_* is the effect of the environment × genotype interaction; and

*ϵ_ijkl_* is the error associated with the *_i_* th environment, *_j_* th replication, and *_l_* th genotype, which is assumed to be normally and independently distributed, with mean zero and homoscedastic

variance *σ*2 (Alvarado et al., 2015).

The annual estimates of genetic gain were obtained as the slope of the regression analysis performed with the BLUPs of each evaluated trait (ordinate) against the year of development/release of the variety (abscissa). For each trait, the absolute and relative rates of genetic gains were presented. The relative rates were calculated by dividing the absolute gain rates by the values for each trait predicted for the beginning of the historical series (De Felipe et al., 2016).

The BLUPs of the joint analysis considering all environments (location × year) where each trait was evaluated were used for the presentation of the results. Simple linear and quadratic regression models were tested to identify whether the rates of genetic gain were constant or discontinuous across the years. The parameters in the linear and quadratic regression models were as follows:


Linear Model:y = a + bx

Quadratic Model:y = a + bx + cx2

where *y* is the dependent variable (agronomic, phenological, and end-use quality traits), *x* is the independent variable (year of variety developed/release), *a* is the intercept, and *b* and *c* are the regression coefficients in different phases of the independent variable (Wang et al., 2016). The analyses were carried out using Meta-R software (Multi Environment Trial Analysis with R for Windows), version 6.0 (Alvarado et al., 2015). Regression analyses to obtain estimates of genetic gain, and construction of graphs were performed using SigmaPlot software, version 11.0. Pearson’s correlation analysis between the BLUPs of the traits was performed using the Genes software (Cruz, 2016). The significance of the regression and correlation coefficients were verified by the *t* test, considering the levels of 5% (*p<* 0.05), 1%(*p<* 0.01) and 0.1% (*p<* 0.001) of error probability.

In order to understand the relationship among grain yield, fodder yield, shoot fly damage, grain mold and protein content, the respective BLUPs and protein content were transformed into principal components and principal component analysis (PCA) was done R (cluster package; Maechler et al., 2014). Based on the year of development/release, the 24 sorghum varieties were categorized under three major improvement periods as varieties developed before the year 2005 (pre-2005), during the period 2005-2015 (2005-2015) and after the year 2015 (post-2015). The PCA biplot was plotted to simultaneously depict the relationship between the varieties (represented as points) and grain yield, fodder yield, grain mold score, shoot fly damage and protein content (represented as vectors).

## Results

### Analysis of variance and mean performance of the sorghum varieties for grain yield and other traits

Combined analysis of variance (ANOVA) across the three test environments during 2018-2020 showed significant (*p*≤ 0.05) mean squares for environments, varieties, and environments × varieties interactions for grain yield, fodder yield, grain mold disease score and shoot fly damage (%) (**Table 3**). The grain yield across the environments ranged from 2290 (Palem 2) to 3866.34 kg/ha (SPV 2579) in PN region, 2775.83 (Palem 2) to 4332.34 kg/ha (SPV 2579) in AT region and 2356.67 kg/ha (Palem 2) to 4010 (SPV 2579) kg/ha in MH region and in overall performance in all the three environments, the grain yield ranged from 2474.17 kg/ha (Palem 2) to 4069.55 kg/ha (SPV 2579). Overall, the mean yield observed across the environments in 24 sorghum varieties was 3459.76 kg/ha with the highest recorded in AT region (3697.64 kg/ha) (**Table 4**).

**Table 3:** Mean squares of grain yield, fodder yield, grain mold score and shoot fly damage (%) of sorghum varieties developed during 1990-2020 across three environments during 2019-2020.

Source of variation	Degrees of freedom	PN region	AT region	MH region
**Grain yield (kg ha^-1^)**				
Environment	2	20837829.78**	26990404.5**	23667583.37**
Replications within Environment	6	49605.47	999644.61**	1127027.77**
Varieties	23	2070840.22**	1825437.98**	2646065.22**
Environments Vs. Varieties	46	582423.81**	586747.92**	514512.68**
Pooled Error	354	7988.67	59300.95	85959.74
**Fodder yield (kg ha^-1^)**				
Environment	2	803286393.68**	84027192.45**	836584747.62**
Replications within Environment	6	23626070.33	46681773.33	38026579.43
Varieties	23	7395982.12*	7063155.3*	74950939.18*
Environments Vs. Varieties	46	21571629.49*	2557349.34*	14328856.01*
Pooled Error	354	800883.74	2373649.48	2220007.46
**Grain mold score**				
Environment	2	231.63**	171.54**	165.36**
Replications within Environment	6	8.90	6.33	8.37
Varieties	23	30.98*	24.15*	28.75*
Environments Vs. Varieties	46	16.19*	16.33*	19.58*
Pooled Error	354	0.60	3.34	0.83
**Shoot fly damage (%)**				
Environment	2	7883.45**	5320.96**	7922.02**
Replications within Environment	6	154.30	180.06	114.96
Varieties	23	773.97**	413.11**	629.82**
Environments Vs. Varieties	46	165.85**	165.24*	208.25*
Pooled Error	354	22.98	22.54	21.09

PN, Palem Nizamabad; AT, Adilabad Tandur; MH, Madhira Hyderabad. * Significant at 5% probability level; ** Significant at 1% probability level.

**Table 4:** Mean performance of sorghum varieties for grain yield, fodder yield, grain mold score and shoot fly damage tested in three environments during 2018-2020.

Variety	Palem/Nizamabad				Madhira/Hyderabad				Adilabad/Tandur				Overall Mean			
	GY	FY	GMS	SFD	GY	FY	GMS	SFD	GY	FY	GMS	SFD	GY	FY	GMS	SFD
CSV 15	2418.33	8490.00	7.58	59.43	2708.33	8981.67	7.68	58.87	2786.67	9631.67	7.35	55.82	2637.78	9034.45	7.54	58.04
Palem-2	2290.00	8800.00	7.57	53.77	2356.67	9075.00	8.12	55.22	2775.83	9750.00	7.27	51.97	2474.17	9208.33	7.65	53.65
PSV 1	2373.00	7885.00	6.45	58.33	2530.00	8828.33	7.18	58.43	2824.17	8845.00	5.70	54.52	2575.72	8519.44	6.44	57.09
PSV 56	2635.00	9110.00	6.92	52.98	2901.67	9420.00	6.65	46.87	3088.33	10048.33	6.78	49.83	2875.00	9526.11	6.78	49.89
PYPS 2	3580.00	18670.00	3.08	29.33	3816.67	19620.00	3.27	30.32	3980.00	21876.67	3.68	31.95	3792.22	20055.56	3.35	30.53
PYPS 8	3801.67	16643.33	3.12	23.18	3595.00	16810.00	3.30	27.93	4273.33	18998.33	3.33	27.23	3890.00	17483.89	3.25	26.12
SPV 2110	3074.00	10420.00	5.73	40.37	3149.67	10881.67	5.20	40.50	3414.00	11845.00	5.28	40.72	3212.56	11048.89	5.41	40.53
SPV 2121	3077.33	10908.33	4.15	36.65	3200.00	11128.33	4.37	37.27	3424.00	12793.33	3.67	33.02	3233.78	11610.00	4.06	35.64
SPV 2122	3266.67	12099.00	6.68	38.62	3460.00	12276.67	5.37	39.88	3713.33	13943.33	5.37	37.78	3480.00	12773.00	5.81	38.76
SPV 2178	3100.00	12420.00	6.40	33.33	3105.00	13063.33	6.92	40.85	3501.67	14203.33	7.13	36.28	3235.56	13633.33	6.82	36.82
SPV 2179	3108.33	13523.33	5.03	32.97	3160.67	13105.00	5.15	35.05	3508.33	14896.67	4.82	30.90	3259.11	14000.84	5.00	32.97
SPV 2242	3376.67	13345.00	6.05	32.61	3498.33	13496.67	7.42	32.92	3945.00	15235.33	7.37	34.94	3606.67	14366.00	6.95	33.49
SPV 2243	3535.00	13610.00	5.93	30.02	3603.33	14326.67	6.90	38.10	3943.33	15863.33	5.70	35.03	3693.89	15095.00	6.18	34.38
SPV 2293	3743.33	14268.33	4.55	37.55	3933.33	14558.33	5.52	44.32	4011.67	16806.67	5.00	41.33	3896.11	15211.11	5.02	41.07
SPV 2294	3522.33	14555.00	4.48	43.15	3690.00	14811.67	6.20	43.42	3935.00	16000.00	5.97	39.80	3715.78	15122.22	5.55	42.12
SPV 2437	3395.00	14423.33	5.78	43.52	3516.67	14725.00	4.65	44.37	3420.00	16048.33	4.95	43.25	3443.89	15065.55	5.13	43.71
SPV 2438	3076.67	16288.33	4.88	39.37	3100.00	15003.33	5.33	39.93	3459.00	17718.33	5.43	41.93	3211.89	16336.66	5.22	40.41
SPV 2502	3416.67	16523.33	5.25	43.16	3718.33	16556.67	4.07	47.42	4036.67	17936.67	5.27	40.83	3723.89	17005.56	4.86	43.80
SPV 2578	3686.67	16323.33	4.32	45.32	3840.00	16756.67	5.03	43.20	4249.00	18191.67	4.38	42.88	3925.22	17090.56	4.58	43.80
SPV 2579	3866.33	15593.33	3.43	38.62	4010.00	16553.33	3.40	44.83	4332.33	18843.33	3.67	39.50	4069.56	16996.66	3.50	40.98
SPV 2678	3728.33	16568.33	4.02	42.95	3905.00	16886.67	3.82	46.02	4240.00	18436.67	4.05	41.40	3957.78	17297.22	3.96	43.46
SPV 2679	3606.67	16565.00	4.23	42.93	3686.67	17165.00	3.77	41.13	3926.67	19000.00	4.38	41.83	3740.00	17576.67	4.13	41.97
SPV 2769	3535.00	16586.67	4.58	41.13	3545.00	17553.33	3.82	40.88	3945.00	18723.33	4.32	38.53	3675.00	17621.11	4.24	40.18
SPV 2770	3576.67	16221.67	4.17	42.08	3540.00	17703.33	4.62	43.00	4009.17	18688.33	4.23	43.05	3708.61	17537.78	4.34	42.71
Overall mean	3282.90	13743.36	5.18	40.89	3398.76	14137.64	5.32	42.53	3697.64	15596.82	5.21	40.59	3459.77	14492.61	5.24	41.34

GY, Grain yield (kg ha^-1^); FY, Fodder yield (kg ha ^-1^); GMS, grain mold score; SFD, Shoot fly damage (%).

The fodder yield across the evaluated environments ranged from 7885 kg/ha (PSV 1) to 18670 (PYPS 2) kg/ha in PN region, 8828.34 kg/ha (PSV 1) to 19620 (PYPS 2) kg/ha in MH region and 8845 kg/ha (PSV 1) to 21876.67 kg/ha (PYPS 2) in AT region whereas the overall fodder yield across the three environments ranged from 8519.44 kg/ha (PSV 1) to 20055.56 kg/ha (PYPS 2). The average fodder yields observed in these three environments were 13743.36 kg/ha (PN region), 15596.82 kg/ha (AT region) and 14137.70 kg/ha (MH region) with an overall mean performance 14492.54 kg/ha for fodder yield (**Table 4**).

Among the varieties, with respect to grain mold incidence, PYPS 2 and PYPS 8 showed low overall grain mold disease scores of 3.25 and 3.34, respectively whereas varieties Palem 2 and CSV 15 showed highest overall incidence of 7.64 and 7.53, respectively. Within each environment, both PYPS 2 (PN and MH regions) and PYPS 8 (AT region) performed the best with the lowest grain molddisease score and CSV 15 (PN region), Palem 2 (MH region) and SPV 2242 (AT region) showed highest grain mold incidence. The overall mean performance of the varieties for grain mold incidence seemed similar in all three environments with a mean disease score of 5.23 (**Table 4**).

The shoot fly damage among the 24 sorghum varieties across the three environments in terms of the percentage of plants with dead hearts ranged from 26.12 (PYPS 8) to 58.04 (CSV 15). Both PYPS 8 and CSV 15 showed the lowest and highest percentage of dead hearts in PN region (23.18 and 59.43%), MH region (27.93 and 58.87%) and AT region (27.23 and 55.82%). The overall mean dead heart percentage of all the tested varieties across the environments was 41.33% (**Table 4**).

### Genetic gains in grain yield, fodder yield, grain mold disease score and shoot fly damage (%)

**Figure 1:**
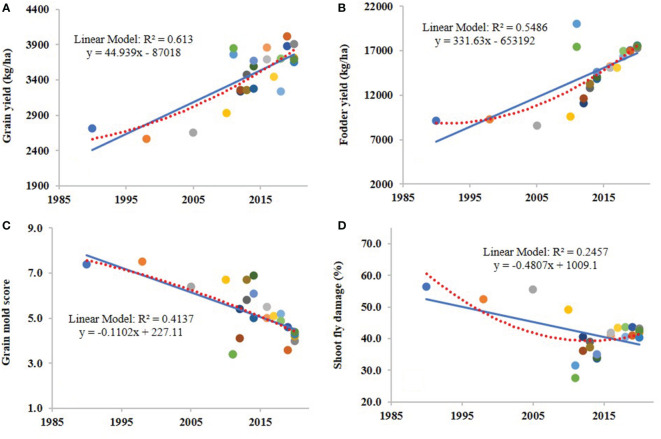
Regressions between the year of development of 24 sorghum varieties and grain yield **(A)**, fodder yield **(B)**, grain mold score **(C)** and shoot fly damage (%) **(D)** for overall environments. Each data point is the best linear unbiased predictor (BLUP) of grain yield, fodder yield, grain mold score and shoot fly damage (%) for a variety in the respective graphs, generated for overall environments.

The results showed significant and positive genetic gains for grain yield and fodder yield in PN, AT and MH regions and for overall environments. Similarly, significant but negative genetic gains were found for grain mold disease score and shoot fly damage (%). The absolute rate of genetic gain for grain yield was 44.93 kg/ha/yr (**Figure 1A**) with relative rate of 1.70% over CSV 15 and 1.29% over the means of all varieties. For fodder yield, the absolute rate of genetic gain for the overall environments was 331.63 kg/ha/yr (**Figure 1B**) and the relative rates were 3.67% over the oldest variety CSV 15 and 2.28% over the mean of all varieties for fodder yield. The BLUP values for grain mold disease score and year of development/release of the sorghum variety were regressed and found that there was a continuous decline in disease incidence in the sorghum varietal development over the last 30 years. The absolute genetic grain for the overall grain mold score in all the environments was -0.11/yr (**Figure 1C**). Overall, the relative rates of genetic gain for grain mold score were -1.46% over CSV 15 and -2.10% over the mean of all varieties. Regression analysis of shoot fly damage (%) against the year of development/release revealed negative rate of absolute genetic gain for the overall shoot fly damage in all the environments was -0.48% per year (**Figure 1D**) and the relative rate was -0.82% over sorghum variety CSV 15 and was over -1.16% over the mean of evaluated varieties.

### Nutritional traits

The protein and fat contents were estimated in 24 sorghum varieties at all the six locations during 2020-21. Significant differences were found among the sorghum varieties for protein and fat contents. However no significant differences were found across the locations. The overall mean protein percentage among the 24 varieties was 10.61% with the highest protein content in PYPS 8 (13.24%) followed by PYPS 2 (13.02%) and the lowest in SPV 2293 (8.7%). The overall mean fat content was estimated at 3.64% with the highest fat content recorded in SPV 2243 (4.20%) and the lowest in SPV 2293 (2.90) (**Table 5**).

**Table 5:** Protein and fat contents in sorghum varieties evaluated at six locations during 2020-21.

Variety	Adilabad		Hyderabad		Madhira		Nizamabad		Palem		Tandur		Overall mean	
	Prot(%)	Fat (%)	Prot (%)	Fat (%)	Prot (%)	Fat (%)	Prot (%)	Fat (%)	Prot (%)	Fat (%)	Prot (%)	Fat (%)	Prot (%)	Fat (%)
CSV 15	9.38	4.33	9.31	4.42	9.42	4.18	9.27	4.21	9.46	3.42	9.22	4.06	9.34	4.10
Palem-2	9.05	4.15	8.95	4.10	9.26	3.96	8.78	4.23	9.33	3.94	9.05	3.95	9.07	4.06
PSV 1	9.82	3.66	9.44	3.78	9.63	3.94	9.42	3.82	9.81	3.62	9.62	3.81	9.62	3.77
PSV 56	9.95	4.12	10.11	3.55	9.78	3.97	10.15	4.38	9.92	3.88	9.88	3.80	9.97	3.95
PYPS 2	12.85	2.98	12.94	3.02	13.16	3.16	13.10	3.18	12.78	2.94	13.26	2.89	13.02	3.03
PYPS 8	12.96	3.09	13.23	3.36	13.30	3.28	13.45	3.16	13.35	3.23	13.16	3.42	13.24	3.26
SPV 2110	10.45	4.10	10.56	3.77	10.38	3.98	10.57	4.32	9.98	3.69	10.26	3.91	10.37	3.96
SPV 2121	10.67	3.92	10.36	3.99	10.59	4.41	10.78	3.81	10.55	3.95	10.58	3.67	10.59	3.96
SPV 2122	10.51	3.81	10.34	3.81	10.52	4.02	10.63	3.92	10.30	3.84	10.52	3.88	10.47	3.88
SPV 2178	10.35	3.68	10.46	3.78	10.38	3.72	10.44	3.45	10.48	3.62	10.33	3.69	10.41	3.66
SPV 2179	10.67	3.60	10.65	3.66	10.63	3.67	10.60	3.52	10.62	3.58	10.54	3.72	10.62	3.63
SPV 2242	10.76	3.23	10.76	3.60	10.88	3.78	10.94	3.58	10.91	3.92	10.84	3.63	10.85	3.62
SPV 2243	10.70	4.31	10.84	4.12	10.92	4.08	10.82	4.18	10.82	4.21	10.75	4.30	10.81	4.20
SPV 2293	8.65	2.92	8.92	2.92	8.38	2.68	8.78	2.66	8.60	3.12	8.84	3.10	8.70	2.90
SPV 2294	9.20	3.65	9.10	3.29	9.31	3.81	9.16	3.48	8.92	3.81	8.98	3.67	9.11	3.62
SPV 2437	10.45	3.29	10.36	3.38	10.28	3.16	10.39	3.65	10.18	3.34	10.32	3.21	10.33	3.34
SPV 2438	10.78	3.92	10.56	3.48	10.63	3.78	10.62	3.96	10.46	3.68	10.38	3.42	10.57	3.71
SPV 2502	10.88	3.98	10.98	3.39	10.92	3.55	10.86	3.84	10.84	3.28	10.82	3.62	10.88	3.61
SPV 2578	11.18	3.80	10.98	3.62	11.21	3.90	10.95	3.72	10.92	3.91	10.96	3.82	11.03	3.80
SPV 2579	11.20	3.38	11.10	3.09	11.38	3.09	11.50	3.18	11.32	3.06	11.22	3.28	11.29	3.18
SPV 2678	11.12	3.46	10.92	3.91	11.22	3.68	11.62	3.77	11.18	3.92	11.26	3.86	11.22	3.77
SPV 2679	11.00	3.54	10.72	3.86	10.78	3.77	10.77	3.09	10.68	3.88	10.82	3.09	10.80	3.54
SPV 2769	11.38	3.28	11.64	3.90	11.29	3.44	11.33	3.60	11.38	4.10	11.22	3.62	11.37	3.66
SPV 2770	10.68	2.92	10.92	3.16	10.80	3.08	10.83	3.24	10.86	3.38	11.16	2.96	10.88	3.12
General Mean	10.61	3.63	10.59	3.62	10.63	3.67	10.66	3.67	10.57	3.64	10.58	3.60	10.61	3.64
CV(%)	7.48	10.47	8.28	16.51	8.12	17.42	5.68	13.73	5.91	11.85	6.42	10.68		
SEM	0.46	0.22	0.51	0.35	0.50	0.37	0.35	0.29	0.36	0.25	0.39	0.22		
SED	0.65	0.31	0.72	0.49	0.70	0.52	0.49	0.41	0.51	0.35	0.56	0.31		
P-Value	0.00	0.00	0.00	0.31	0.00	0.25	0.00	0.01	0.00	0.03	0.00	0.00		
CD(5%)	1.30	0.63	1.44	0.98	1.42	1.05	0.99	0.83	1.03	0.71	1.12	0.63		

**Figure 2:**
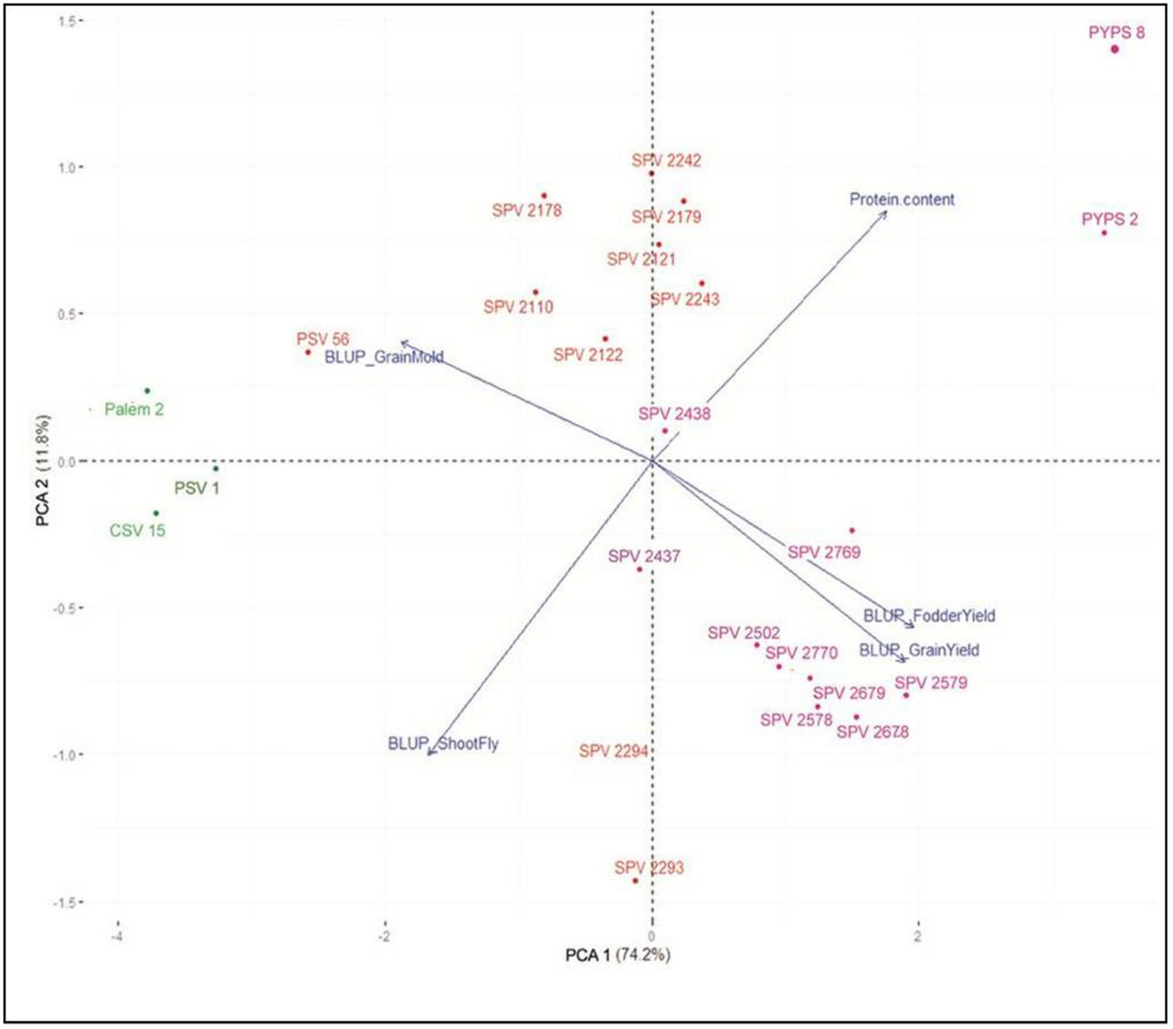
Biplot of the first and second principal components based on the best linear unbiased predictor (BLUPs) for grain yield, fodder yield, grain mold score and shoot fly damage (%) and protein content for a total of 24 sorghum varieties. Data points in green indicate varieties developed before 2005 (pre-2005), red indicate those developed during 2005-2015 and purple indicate varieties developed after 2015 (post-2015).

Using PCA, the five-dimension trait BLUPs were reduced to two dimensions explaining 86.0% of the relationship (**Figure 2**). The PCA biplot showed that the grain yield was positively correlated to fodder yield and was partially correlated to the protein content. Grain and fodder yields were negatively correlated with shoot fly damage and grain mold. Sorghum varieties CSV 15, Palem 2 and PSV 1, developed during the first improvement period (1990-2005), showed high shoot fly damage and grain mold incidence. The sorghum varieties SPV 2578, SPV 2579, SPV 2678, SPV 2679 and SPV 2770 developed during the third period (2015-2020) were localized bottom right with high grain and fodder yields. The yellow pericarp sorghum varieties PYPS 2 and PYPS 8 located at the top far right showed high protein content with tolerance to grain mold.

## Discussion

In this study, a total of 24 sorghum varieties developed during the period 1990-2020 were evaluated in three environments comprising six locations for three years. Significant and high mean squares detected for grain yield, fodder yield, incidences of grain mold disease score and shoot fly damage (%) in three environments indicated that the varieties responded differently across the environments, which seemed to be unique in identifying superior varieties. The presence of significant differences among the varieties for grain yield, fodder yield and incidence of grain mold and shoot fly indicated the existence of genetic variability among the varieties developed during 1990-2020.

Three varieties SPV 2579, SPV 2678, SPV 2578 in AET-I stage during 2018-19 consistently showed highest mean grain yields whereas PYPS 2 (released in 2020) and PYPS 8 (AET-II stage and being proposed for release) showed highest mean fodder yields. Three oldest varieties used in the study (CSV 15, PSV 1 and Palem-2) had low grain and fodder yields. Overall, the grain yield increased substantially with the development of improved varieties. This is in agreement with other findings such as in chickpea, which also reported a significant increase in grain yield of chickpea varieties over the old ones (Tadesse et al., 2018). In another similar study, better seed yield of newly developed soybean cultivars over the first old variety was reported (Demissew, 2010). This gives an insight into possible future opportunities to exploit the genetic potential of the crop for enhanced sorghum production.

In general, the genetic yield gain for grain yield in sorghum has been reported to be significantly lower than in other major field crops (Pfieffer et al., 2019). In absolute terms, we have found that the genetic gain in grain sorghum (44.93 kgha^-1^yr^-1^) is similar to soybean (43 kg/ha/yr) (De Felipe et al., 2016) but it was lower than in maize (55-75 kg/ha/yr) (Apraku et al., 2022). In sorghum, we have reported much higher genetic gains for grain in varieties compared to that of 8.7 kg/ha/yr in hybrids of Argentina (Gizzi and Gambin, 2016) and 8 kg/ha/yr in both hybrids and inbred lines of USA (Pfeiffer et al., 2018). The low genetic gain in the former attributed to irregular breeding efforts for quality improvement (tannin concentration and fat content) could mean that the continuous sorghum breeding efforts in India were successful as evident by high genetic gains and also that breeding for stress-tolerance (grain mold and shoot fly tolerance)was more fruitful compared that of quality improvement (Gizzi and Gambin, 2016). The low genetic progress rates estimated by Pfeiffer et al. (2018) attributed to limited genetic diversity within their crop breeding program implied the high genetic diversity within the Indian sorghum breeding program.The 24 sorghum varieties were developed through pedigree method of selection involving hybridization between landraces and elite breeding lines, resistant germplasm lines and improved varieties. These lines were from the interior parts of southern India at the regional level, ICAR-IIMR and ICRISAT at the national and international level respectively, which might have contributed to the high genetic variability, as evident from the high mean square values. This in turn might have facilitated accelerated genetic gains from selection for improvements in grain yield and other traits with further scope for identification of sources of genetic variability for development of improved genotypes. This, however, is in contrast to the findings of Rakshit et al. (2014) who have reported very high genetic gains in grain yield (rainy season varieties) at 90 kg/ha/yr (which is double that of our study) from 1970- 1980 and after that until 2009, there were non-significant changes with the grain yield gain thus reaching a plateau. They also reported no significant changes in fodder yield for rainy season varieties compared to estimated gains of 331.63 kg/ha/yr in this study. This indicated successful varietal improvement for dual-purpose sorghum suitable for rainfed conditions during 1990s through 2020s where breeders gave equal importance to grain yield and fodder yield (Ashok Kumar et al., 2011; Reddy et al., 2012) compared to the hitherto efforts in the rainy season which were focused mainly on grain yield (Audilakshmi et al., 2011). Further according to Rakshit et al. (2014), the genetic gains for grain yield in post-rainy season sorghum varieties were compromised because the breeding focus was on developing dual purpose sorghum and low genetic variability, predominantly representing *durra* races (Sajjanar et al., 2011; Rakshit et al., 2012), has limited the genetic progress in post-rainy reason sorghum. The wider genetic variability representing *caudatum*, *bicolor*, and their intermediary races (Aruna and Audilakshmi, 2008) available in the rainy season sorghum might have also contributed to the high genetic gains for grain and fodder yields estimated in our study. Besides, in our study there was no indication of yield potential plateau in sorghum varieties unlike the findings of Rakshit et al., 2014 implying that further improvement is possible to increase the yield and to further exploit the yield potential of existing varieties.

Because the genetic gain measured in kilogram per hectare per year is positively associated with the quality of the environment (Rinker et al., 2014), it is important to use the relative genetic gain as a way to compare productive systems that have different starting points or average yields (Slafer and Andrade, 1991), which is mostly the case for genetic gain studies in long term breeding programmes across the world. In our study, the relative genetic progress made in grain yield (1.70%) from sorghum breeding in India were higher than the progress made in seed/grain yields of other crops like groundnut (1.89%) (Hagos et al., 2012) and haricot bean (3.24%) (Bezawuletaw et al., 2006) and lower than barley (1.34%) (Fekadu et al., 2011), soybean (1.1%) (De Felipe et al., 2016), (0.33 to 0.77%) (Milioli et al., 2022) and chickpea (0.57%) (Tadesse et al., 2018). In maize, varying relative genetic gains reported at 0.5% (Curin et al., 2020), 0.62% (Liu et al., 2021), 0.83% (Di Matteo et al., 2016) were due to differences in the growth environments in their studies and the types of cultivars (single and/or double cross) etc. In sorghum, however, there are no reports of genetic gain expressed relative to the predicted yield for the oldest release year considered in the experiment.

To our knowledge, this is the first study to report negative genetic gains for grain mold score and shoot fly damage (%) in sorghum indicating the genetic progress made in improving tolerance to both these stresses. Two dual-purpose sorghum varieties PYPS 2 and PYPS 8 showed tolerance to grain mold coupled with high grain and fodder yields. Similar findings were reported by Kumar et al. (2021). The positive correlation between the grain yield and fodder yield and their negative correlations with shoot fly damage and grain mold incidence suggested that the genetic gains in grain and fodder yields might be due to improved tolerances to the shoot fly and grain mold disease. In all the varieties and across the environments, shoot fly incidence (percentage dead hearts) was above 25% (except for PYPS 8 in PN region). Above 50% dead heart percentage was observed in varieties CSV 15, Palem-2, PSV 1 and PSV 56, all four of which were released during 1990-2010. Grouping the varieties based on the period of development in the PCA biplot also corroborated the same as indicated by the low grain and fodder yields of pre-2005 varieties CSV 15, Palem 2 and PSV 1 with high shoot fly damage and grain mold incidence. It was during this period that the sorghum research was re-oriented to include tolerance to shoot fly as a mandatory criterion for the development and release of sorghum varieties at the national level, the result of which was evident with moderate resistance observed in shoot fly incidence among the varieties developed post-2010. Currently, at the national level screening, the entries must show maximum shoot fly damage (%) of 30-35% or less to be considered for further evaluation. However, none of the varieties tested in this study were found resistant to shoot fly implying more intensive breeding efforts are required toward shoot fly resistance.

While the significant genetic progress in sorghum yield and other traits in this study corroborate the success of breeding efforts to develop dual-purpose sorghum with grain mold tolerance, it is very critical that the old varieties are replaced by the new ones with higher potential productivity in achieving continuous genetic gains (Yadav et al., 2021). Breeding materials should be diversified with new germplasms, lines and varieties replacing the old ones for developing good varieties for dual purpose. Pre-breeding with wild sorghum relatives will help in the development of diverse new varieties for higher genetic gains (ACIRP on Sorghum, 2022). New landraces need to be utilized for broadening the genetic base of the material. In this study, two yellow sorghum varieties PYPS 2 and PYPS 8 had overall low shoot fly and grain mold incidence with high grain and fodder yields. These were previously identified as best dual-purpose varieties with stability (Kumar et al., 2021). Even though the PYPS 2 was released in 2020, the variety found wide acceptance among the farmers and high consumer preference even before its release because of high grain mold tolerance, early maturity, high protein content and good flatbread making quality (Jaisimha, 2019). PYPS 8 is another high protein-containing sorghum variety currently in AET-II evaluation stage. Both PYPS 2 and PYPS 8 were developed from a pool of 11 parental landraces, which could offer potential new sources genes for high grain and fodder yields and stability (Kumar et al., 2021).

Further, fast replacement of varieties with new ones allows farmers to exploit more fully the genetic gains from plant breeding (Singh et al., 2020). In the United States, the development of a new maize hybrid takes an average time of 6 years and remains in the seed chain for three to four years (Singh et al., 2020). However, in the Indian context, the average time for development of a sorghum variety through pedigree method takes around 10-15 years and usually, there is a delay of 4 to 6 years between the official notification of a variety and its commercial cultivation (Witcombe et al., 1998). The high-yielding varieties under cultivation in India are about 15 years older than in a very efficient system and even halving the gap would result in 7-15% increase in yield for farmers growing these modern HYVs assuming a 1-2% genetic gain per annum. By growing older varieties, farmers are missing out on the benefits of many years of genetic gains from the breeding programs which are meant to serve them but are insufficiently linked due to dysfunctional varietal release and seed systems (Atlin et al., 2017). In developing countries like India, varieties used by the farmers should not be older than 10 years. These gains are transferred to the field through concerted efforts from regulatory bodies, breeding organizations, seed companies and national seed systems.

In our study, we used single-trait BLUP which is the most widely employed selection method to estimate the genetic gains in sorghum. When traits are correlated and complex, genetic evaluation using multi-trait BLUP can be more efficient (Viana et al., 2010). Recently, de Souza et al. (2019) showed that multi-trait BLUP predicted higher selection gain and demonstrated its efficiency in the genetic selection of grain sorghum for flowering time, plant height and grain yield. We found two protein-rich varieties PYPS 2 and PYPS 8 as high-yielding dual-purpose sorghum varieties, both of which were previously reported for their best and most stable performance in drought-prone environments (Kumar et al., 2021). Both PYPS 2 and PYPS 8 also contain high zinc content (Kumar et al., 2021), which is positively correlated with protein (Badigannavar et al., 2016). These could be further evaluated using Multi-trait BLUP to identify superior genotypes with improved nutritional traits in sorghum.

Sorghum is one of the cheapest sources of energy and micronutrients in India and Sub-Saharan Africa. Biofortified sorghum is a cost-effective and sustainable solution for combating micronutrient deficiencies where sorghum provide more than half the dietary micronutrients to the low-income group, particularly in rural India, where both physical and economic access to nutrient-rich food is limited (Rao et al., 2006, Rao et al., 2010). Keeping in view the wide-spread nutritional deficiencies, improving grain nutritional traits (protein, iron and zinc) has been a recent addition to the breeding objective in sorghum. This study found significant differences in protein content among the 24 sorghum varieties with highest protein recorded in PYPS 2 and PYPS 8, both of which were earlier reported as high protein-containing genotypes (Kumar et al., 2021). High variability in protein ranges between 9-11% in genotypes grown under different conditions (Elbashir et al., 2008; Ahmed et al., 2014), 10-11% in germplasm collections (Weckwerth et al., 2020), 9-14% in lines developed from landraces (Kumar et al., 2021), 6-13% in landraces (Abdelhalim et al., 2021) has been reported previously suggesting the feasibility of genetic enhancement of protein. However, the key challenges associated with the use of sorghum as protein-rich food are low protein digestibility and poor protein quality (low lysine), which reduce its nutritional value especially for those who rely on sorghum as a staple food source. Breeding efforts to improve these were successful with *hd* (high digestible) proteins transferred into well-established sorghum hybrids resulting in improved protein digestibility by 25-40% (Teferra et al., 2019). More recently, clustered regularly interspaced short palindromic repeats (CRISPR) gene-editing technique was used to develop sorghum variants with improved protein quality (increased lysine content) and digestibility (Li et al., 2018) thus presenting a new opportunity for more rapid and precise sorghum improvement. Future sorghum genetic improvement programs should focus on routinely evaluating the genotypes for protein digestibility and quality in addition to testing for nutritional contents to ensure sorghum becomes and remains a competitive crop with a maximum positive impact on human health particularly in the arid and semi-arid regions of India and Sub-Saharan Africa.

## Conclusion

The present study reports high overall genetic gains of 44.93 kg/ha/yr and 331.63 kg/ha/yr for grain yield and fodder yield respectively, over the first released sorghum variety CSV 15. Under the present climate change scenario, two dual-purpose, grain-mold tolerant and protein-rich varieties, PYPS 2 and PYPS 8, derived from superior landraces, are identified for cultivation in drought-prone regions of India. The study corroborated the successful breeding approaches employed towards sorghum varietal improvement in India. The absence of yield plateau implies further possibility for yield improvement by exploitation of the existing varieties, landraces and wild sorghums through advanced genomic tools and precision selections to accelerate the breeding of sorghum in India and ultimately contribute to global nutritional, food and feed security

## Data availability statement

The original contributions presented in the study are included in the article/**Supplementary Material**. Further inquiries can be directed to the corresponding author.

## Author contributions

MN, VR, MS and KK designed and carried out the experiments. MN, VR, MG and AD analyzed the data. VR, MS and KK conducted the field experiments with logistical support by GK and RJ. MV, RV and MG wrote the draft manuscript. All authors contributed to the article and approved the submitted version.
