# Supplementary material for: India’s rainfed sorghum improvement: Three decades of genetic gain assessment for yield, grain quality, grain mold and shoot fly resistance
Source: Front Plant Sci. 2022 Dec 19;13:1056040. doi: 10.3389/fpls.2022.1056040 (PMC9806348; doi:10.3389/fpls.2022.1056040)
Supplement: Supplementary file 1 [file DataSheet_1.docx]

Supplementary Figures


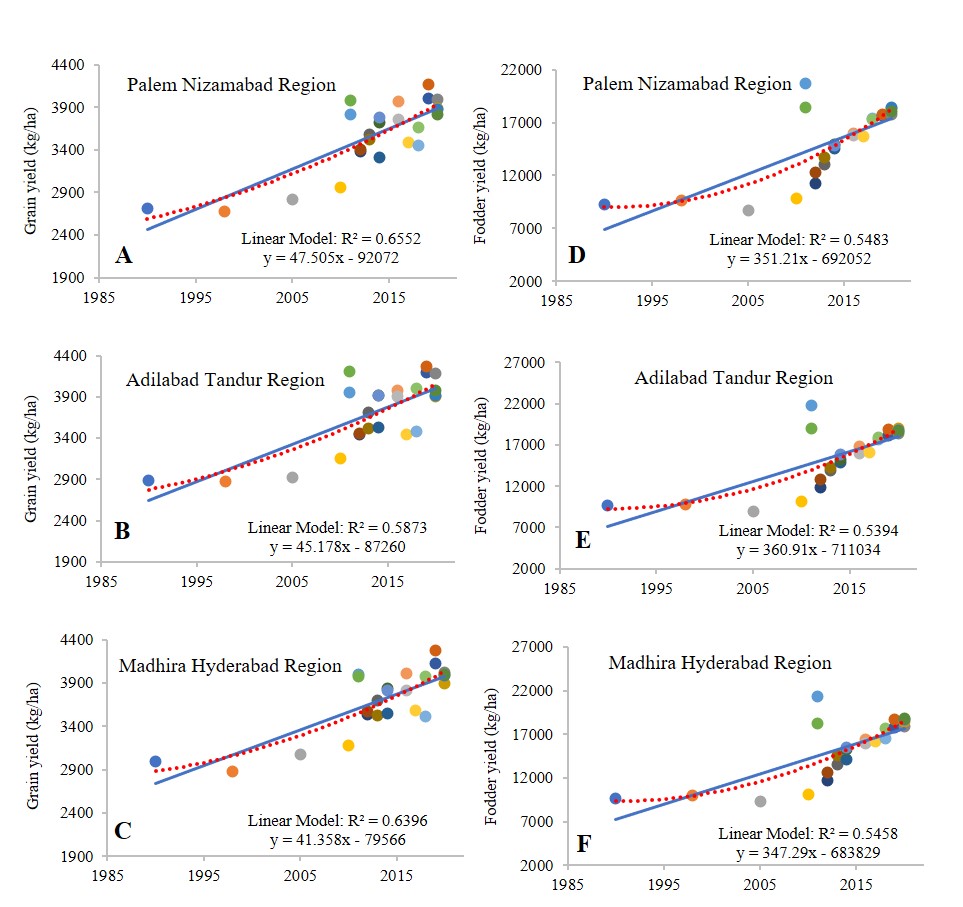


Supplementary Figure 1. Regressions between the year of development of 24 sorghum varieties and grain yield for PN region (A), AT region (B) and MH region (C) and fodder yield for PN region (D), AT region (E) and MH region (F). Each data point is the best linear unbiased predictor (BLUP) of grain yield, fodder yield, grain mold score and shoot fly damage (%) for a variety in the respective graphs, generated for three environments.


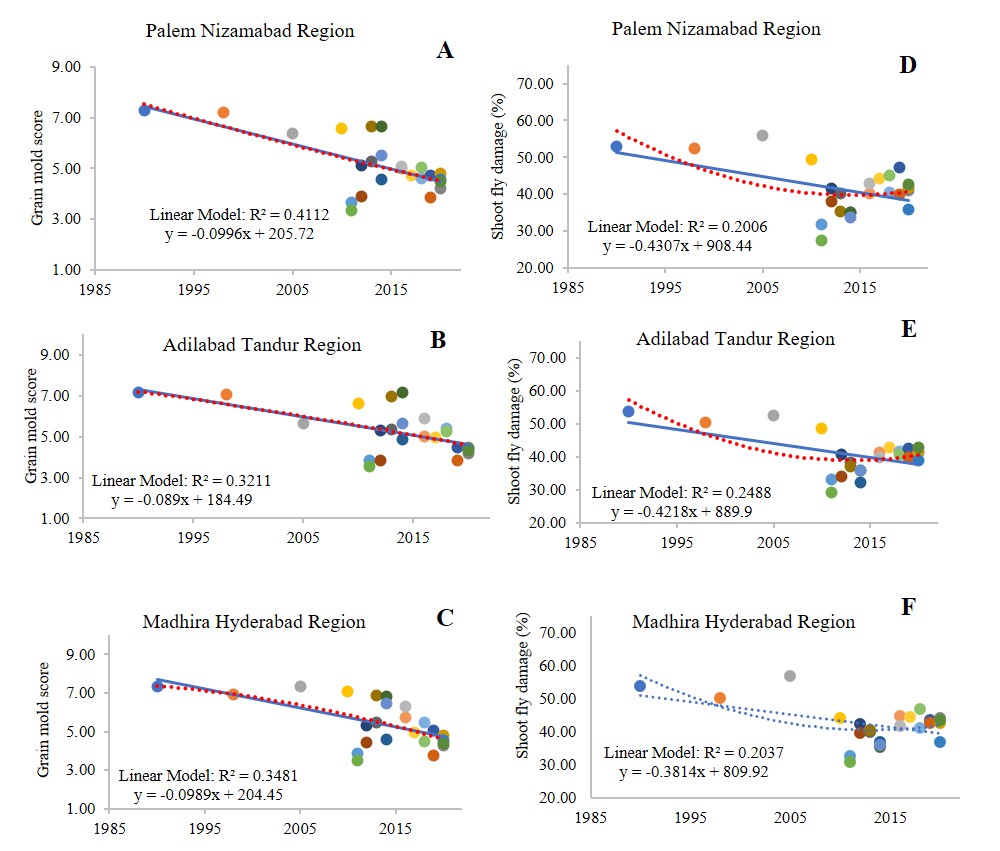


Supplementary Figure 2. Regressions between the year of development of 24 sorghum varieties and grain mold score for PN region (A), AT region (B) and MH region (C) and shoot fly damage (%) for PN region (D), AT region (E) and MH region (F). Each data point is the best linear unbiased predictor (BLUP) of grain yield, fodder yield, grain mold score and shoot fly damage (%) for a variety in the respective graphs, generated for three environments.
